# Messy Chemistry and the Emergence of Life

**DOI:** 10.3390/life16020186

**Published:** 2026-01-23

**Authors:** Alberto Vázquez-Salazar, Ranajay Saha

**Affiliations:** 1 Departamento de Bioquímica, Centro de Investigación y de Estudios Avanzados del Instituto Politécnico Nacional, Av. IPN 2508, San Pedro Zacatenco, Gustavo A. Madero, Ciudad de México 07360, Mexico; 2 Department of Chemical and Biomolecular Engineering, University of California, Los Angeles, CA 90095, USA

**Keywords:** messy chemistry, origin of life, prebiotic chemistry, LUCA, chemical evolution

## Abstract

Chemical complexity is not a nuisance to be minimized in origin of life research, it is an enabling condition. This second edition of the Special Issue on the Origin of Life in Chemically Complex Messy Environments gathers contributions that embrace multicomponent mixtures, dynamic geochemical settings, and nonequilibrium processes. The papers collected here survey surface hydrothermal routes to reactive nitriles, groundwater evolution of alkaline lakes, and transition metal sulfide-driven amino acid and amide formation without cyanide. They report one pot nucleoside and nucleotide synthesis from formamide over cerium phosphate, review non aqueous organophosphorus pathways, and probe peptide rich mixtures and formose type networks under serpentinization associated minerals. The issue also advances conceptual frameworks, including atmospheric photochemical signatures for biosignature discrimination, the role of chiral mineral surfaces in enantioseparation, and computational simulations of the origin of LUCA. Together, these studies position messy chemistry as a crucible that turns chemical diversity and environmental heterogeneity into routes toward organization and function.

## 1. Messy Beginnings

The origin of life did not emerge from tidy, isolated reactions in pristine laboratory flasks. Rather, it likely arose amid chemically complex, messy environments on the early Earth, amid chaotic mixtures of diverse interacting molecules. Dynamic settings, from impact heated hydrothermal systems to evaporating alkaline lakes, offered a rich tapestry of molecules, minerals, and physical gradients. This second edition of the Special Issue on the Origin of Life in Chemically Complex Messy Environments builds on the momentum established by the first edition, which consolidated diverse contributions across disciplines [1]. The present volume extends that trajectory by foregrounding experiments and models that work with, not against, chemical heterogeneity.

Beyond acknowledging complexity, messy chemistry reframes one of the central questions of origin of life: in multicomponent mixtures, function can emerge from interactions among many species rather than from a single privileged pathway. In line with this, systems chemistry provides a unifying perspective, where selection, feedback, and network connectivity channel reaction fluxes and stabilize structure in far from equilibrium conditions [2]. Recent automation and artificial intelligence tools add complementary power, enabling long duration, data rich, and algorithm-guided searches through vast chemical spaces that messy systems naturally generate [3,4]. Interfacial processes, cycling regimes, and spatial gradients become drivers rather than confounders, with nonequilibrium forcing that can bias ensembles toward organization.

## 2. Geochemical and Planetary Contexts

Several contributions presented here place messy chemistry in specific planetary contexts. Lyons et al. outline how isotopic ratios of key atmospheric gases—carbon, nitrogen, sulfur can help discriminate biological from abiotic processes in exoplanet atmospheres, a framework that could connect atmospheric observables with prebiotic chemistry and early metabolisms [5]. Rimmer and Shorttle further demonstrate that surface or shallow hydrothermal settings can supply nitriles and isonitriles, which are key reactive intermediates for nucleotide and amino acid chemistry, directly from geologically plausible gases and graphite rich regions of the Hadean crust generated from giant impacts [6]. Similarly, Tutolo and colleagues analyze how groundwater flow can evolve alkaline lake chemistries over time, creating dynamic regimes of concentration, pH, and ionic composition that promote prebiotic synthesis and earliest metabolic pathways [7].

## 3. Novel Prebiotic Pathways in Complex Mixtures

At the molecular scale, messy does not imply uncontrolled. The present volume shows plausible prebiotic pathways for synthesizing life’s building blocks. Gilboa and coauthors report light assisted, one pot synthesis of nucleosides and nucleotides from formamide in the presence of cerium phosphate, which acts as photocatalyst and phosphate donor in a compact route that exploits photochemical energy input in the first step for nucleobase formation [8]. The work shows the possible role of heterogeneous photocatalysis in forming life’s building blocks like RNA and DNA. In addition, demonstrating a cyanide-free route to peptide precursors, Seitz and colleagues show that acetylene, ammonia, and carbon monoxide can yield amides and amino acids in aqueous solution under transition metal sulfide catalysis, a pathway that broadens prebiotic feedstocks beyond classical HCN-based schemes [9]. To address the phosphate availability problem on early earth, Gull and coauthors show organophosphorus synthesis in non-aqueous and semi-aqueous prebiotic solvents from reduced phosphorus sources under mild heating conditions, thereby expanding the solvent and redox landscape for prebiotic phosphorylation [10].

## 4. Reaction Networks, Emerging Polymers, and Mineral Coupling

Messy mixtures can also support rich network behaviors, including autocatalysis, selection, and phase coupling. Omran and coauthors probe serpentinization associated mineral catalysis in a protometabolic formose system, highlighting how alkaline hydrothermal minerals can direct carbohydrate networks toward complexity [11]. Additionally, Shalayel and coauthors report straightforward routes to complex mixtures of thiol rich peptides, relevant to redox active functional ensembles where molecular diversity followed by selection were more crucial for the emergence of life [12]. Both works exemplify how mineral surfaces, pH gradients, and reactive fluxes reshape network topology and channel product distributions.

## 5. From Chemical Diversity to Evolutionary Scenarios

Messy chemistry also informs evolutionary narratives. For example, Tang proposes a computational framework for the origin of LUCA, exploring how environmental fluctuation and selection over networks could have shaped proto metabolic organization and information flow [13]. Moreover, Bielski and Tencer analyze the possibility of enantioseparation of racemic ribose on chiral mineral surfaces formed by nucleobase adsorption, linking geochemistry, adsorption physics, and chiral amplification in a way that bridges plausible prebiotic asymmetry with later biochemical homochirality [14]. Overall, these studies do not seek single path inevitability, but they show how classes of messy environments can bias ensembles toward biological order.

## 6. Conclusions and Future Directions

Collectively, these contributions reinforce that messiness was a crucible—not a constraint—for the emergence of life. From one pot nucleotide formation in formamide-rich mixtures, to amino acid and amide synthesis from simple gases under sulfide catalysis, to organophosphorus chemistry in non-aqueous media, the studies in this volume show how diverse inputs and dynamic settings can be harnessed rather than sanitized [8,9,10]. Network level behaviors recur as a theme, with mineral templating, cycling regimes, and spatial gradients guiding transitions from chemical diversity to functional assemblies [11,12,13,14]. Conceptual frameworks that tie atmospheric photochemistry to biosignature inference push this integration up to planetary scales [5].

Challenges ahead include analytical tractability in multicomponent mixtures, comparative studies across geochemically realistic parameter spaces, and models that couple reaction networks to transport, phase behavior, and energy fluxes. Instrumented, long duration platforms that integrate automation, closed loop optimization, and high-resolution analytics will be essential for mapping large, rugged chemical landscapes [3,4]. Field-linked experiments that test hot spring cycles, shoreline concentration gradients, and evaporative microenvironments can connect laboratory control with natural complexity, building on evidence that interfacial and cycling processes drive selection in prebiotic ensembles [15]. The cross fertilization of geochemistry, chemical physics, data driven exploration, and theory, evident in this issue, points toward an origin of life that emerges as organized complexity from primitive chaos, with messy chemistry as both starting point and the organizing principle.

Finally, the first edition of this Special Issue was consolidated as an MDPI Book, an outcome that reflects community engagement across disciplines and that encouraged this expanded second edition [1]. The trajectory continues, toward broader parameter sweeps, deeper planetary context, and tighter feedback between theory, field analogs, and laboratory automation.
